# Odontological Society of Pennsylvania

**Published:** 1889-05

**Authors:** 


### Reports of Society Meetings.

### ODONTOLOGICAL SOCIETY OF PENNSYLVANIA.

THE REGULAR MEETING OF THE ODONTOLOGICAL SOCIETY OF PENNSYLVANIA WAS HELD SATURDAY EVENING, JANUARY 5TH, 1889, AT THE HALL, THIRTEENTH AND ARCH STREETS.

President Kirk in the chair.

A paper on

### THE ADVANTAGES OF COTTON AS A ROOT CANAL FILLING.

WAS PRESENTED BY JOSEPH HEAD, D. D. S., PHILADELPHIA.

At the tenth anniversary meeting of this Society, held in December, not a single objection was raised against cotton root-canal fillings, which, in my opinion, did not equally apply to those of gold, oxyphosphate of zinc, or gutta-percha.

It was said that cotton was prone to absorb fluid from the tissues around the apex of the root, which fluid, undergoing decomposition, was likely to cause alveolar abscess.

For the present, this statement shall remain undisputed, although I hope, before the reading of this paper is finished, to prove to your satisfaction that such is not the case.

It is unnecessary for me to describe the method of treating single rooted teeth, such as the canines and incisors. Since the orifices of the canals are evident and the line of direction straight, enlarging, cleansing, and thorough adaptation of the root filling is extremely easy. In fact, so few difficulties are these teeth likely to present, that the student may be quite as successful in their treatment as the expert.

Therefore, let us consider only the teeth which are difficult to quiet, feeling well assured that in learning to apply the proper therapeutic skill to these, we shall at the same time be mastering the methods to be used in combating all the lesser evils.

In pursuance of this idea, I would invite you to consider with me the treatment of bicuspids and molars. Yes, the manner of dressing and filling molar roots shall be the subject of discussion, for these as a class are likely to give more trouble of a peridental nature than all the rest of the teeth combined.

Why? Because this class, not only being larger than any of the others, contain forty canals, each capable of causing an alveolar abscess, while all the other teeth can muster only twenty-four. And because a tooth containing two or three canals is two or three times as likely to give trouble as a tooth which contains but one. With this hasty explanation let us now proceed to inquire if gold cement or gutta-percha can justly be said to make a permanent or perfect filling.

At the late anniversary an eminent practitioner told me that he first measured the end of the canal and then filled the apical end solidly with gold. So great was his regard for his method, that he did not approve of cotton fillings.

I wondered how he would manage to do this to the anterior buccal canal of a superior molar. I should have been satisfied had he told me how he would measure and fill solidly, with gold, the flat roots of lower molars, the canals of which so frequently sub-divide, leaving between the two branches a translucent plate of cementum.

It occurred to me, that while enlarging these sufficiently for the complete accomplishment of his design, that there would be danger of boring into the alveolar process, the blood thereby spoiling the perfect adaptation of the gold. Should his practice become general, it would probably work great injury to all roots containing minute, flat, or tortuous canals. As in so treating them would he not, in all probability, either perforate the root, or leave untouched a large portion of the canal, into which it would be impossible thoroughly to pack the gold? There being no method of venting the tooth, would he not incur the risk of alveolar abscess with all its horrors?

Another experienced practitioner said that after sterilizing the canals, he pumped into them gutta-percha dissolved in chloroform, and then packing the root with gold, made, as he said, a filling so impervious to fluids from without, that alveolar abscess was an impossibility; or, at least, so improbable that its occurrence was not to be considered.

This method at first sounded plausible, but, after deliberation, it appeared to me that his materials were not equal to good gutta-percha. The gold in his case, occupying the position of an inert mass, could only serve to block the canals should trouble arise. While all the responsibility of excluding noxious fluids was given to gutta-percha, the real strength and toughness of which had been destroyed by the chloroform.

It is not necessary to prove to you that dental gutta-percha is porous, that is, it does not make a water-tight filling. These facts are too well known, but if a skeptic be present, a single sniff at the gutta-percha found around most of the pivot teeth set in that material will fully satisfy his doubts as to its porosity ; while the fact that it leaks has been proven again and again by the glass tube and analine ink test.

Therefore, if dental gutta-percha be not a bar to the fluids of the blood, and if it cannot be denied that it absorbs decomposable liquid, and not being a vehicle which will combat putrefaction, how can it be called a perfect or permanent filling ?

How is it possible to suppose that a root so filled can be granted immunity from gases which cause alveolar abscess. How can it be believed that this much lauded material is even as good as the despised cotton, which does not pretend in itself to exclude moisture, but acts solely as a vehicle for retaining medicine which will keep out fluids and at the same time prevent the development of putrefactive germs still lingering within the canals.

Dental gutta-percha not only offers free entrance to fluids of the blood, but, being inert, affords them an excellant harbor where they may multiply with the greatest rapidity.

But perhaps some of you at this moment are anxious to rise and speak as follows : "How is this, my young friend? Gutta-percha porous? Consider that the Atlantic Cable is wrapped in gutta-percha, and if a single drop of water should leak into it so as to touch the metal, the cable would be useless." To this I should reply as follows: "Respected sir, I am not speaking of pure gutta-percha, but of the material used by dentists. Pure gutta-percha has not sufficient body to be used in our profession. Oxide of zinc, which is extremely soluble, is usually mixed with it, and when the zinc is dissolved, the gutta-percha becomes like a sponge."

But granting that the gutta-percha be mixed with an insoluble powder, still gutta-percha, being a leaky material, allows the fluids to enter beside the earthy particles of the powder, and is, therefore, only slightly less pervious than when oxide of zinc is used. Should base-plate, which is almost pure gutta-percha, be used, the same objection may be made, as it is also mixed with a powder and presents the insurmountable difficulty of not making a water-tight joint, thereby incurring the risk of alveolar abscess.

What has just been said of gutta-percha is equally applicable to cements. True, they make a tight joint ; but then they are so porous that they have all the harmful properties attributed to gutta-percha, and also an important additional one, namely : that it cannot be removed, and, if trouble arise, relief is impossible except by counter-irritation or puncture of the alveolus.

Counter-irritation, in most cases, seems like trying to put out the sun with a squirt-gun; for, if the abscess has to run its course, the slight success which a dentist's conscience permits him to attribute to his efforts is often unperceived by the patient. While puncturing the alveolus is not only a severe operation, but too often unattended with any other result than an increase of pain, failure is either due to the fact that pus has not yet formed, or that the pus cavity has been missed by the drill. While we are on this subject I should like to ask two questions about alveolar abscess.

Does not the patient suffer prolonged and excruciating pain before pus is formed? Before the pus is generated in a quantity sufficiently large to be successfully pierced, has not the patient endured by far the greater part of the pain which would naturally fall to his lot?

Gentlemen, I think you will answer both these questions in the affirmative, and, therefore, taking into account the uncertainty and discomfort occasioned by the drilling, the expediency of relying on this operation as a means of relief can be questioned with some appearance of logic. Therefore, it would seem that the dentist who accepts these facts, and yet, in a case of simple treatment, knowingly uses an irremovable canal filling which has the defects above explained, wilfully subjects his patients to the risks of not improbable alveolar abscess with all its accompanying horrors.

Gutta-percha, in roots where pivots are placed, can, as a rule, be readily removed from the canal, should venting be necessary. Hence, this material should always be preferred whenever its use will ensure sufficient stability; and where cement is necessary to render venting easy, a small piece of gutta-percha, or a small pellet of cotton dipped in an antiseptic, should first be placed at the apical end.

The advocates of the so-called permanent root fillings, gold, gutta-percha, and cement, claim that these agents absolutely exclude putrefactive germs. And, moreover, the root being sterilized previous to filling, that these materials so protecting it will soon make alveolar abscess one of the lost diseases. Are these statements borne out by facts? Do not these fillings contain all the disadvantages which have been attributed to cotton? It is said that cotton is not water-tight. Are the other materials water-tight? It is said that it absorbs fluid which, decomposing, gives rise to irritating vapors. Do not gold, gutta-percha, and cement also give rise to irritating vapors?

But the great advantages of cotton dressings have not been justly rated.

Firstly, it can be easily removed. Secondly, it can be thoroughly permeated with medicaments which will not only destroy septic matter, but prevent its entrance.

In reply to the first of these statements, our learned friends claim that alveolar abscess, in teeth treated by their method, is so improbable that the removal of the filling is never necessary, external treatment meeting every requirement.

Well brother practitioners! if, after the review which has just been made of the obvious imperfections of gold, gutta-percha and cement as canal fillings, these gentlemen still adhere to their opinion, it must be confessed that I am unable to find any argument which might convert them.

Therefore, without further discussion of the great advantage which cotton has in its easy removal, I will pass at once to the consideration of cotton as a medium for antiseptic medication.

It has been said that "The medicament evaporating, leaves the cotton unguarded."

Gentlemen, have you ever heard of carbolized cosmoline? and will you kindly inform me what is its daily rate of evaporation?

It is with carbolized cosmoline that a cotton dressing should be soaked.

The late Dr. H. A. Randolph boiled a frog's foot in pure cosmoline in order to destroy any putrefactive germs which might remain, and then allowed it, covered by the paste, to stand for an indefinite time. Week after week the foot stayed unchanged, the experiment proving that cosmoline is aseptic in the highest degree, and that a sterilized body placed in it will remain intact so long as it is covered.

Therefore, gentlemen, it seems to me strongly indicated by the majority of facts; for these experiments can be verified, that, while gold, guttapercha and cement have all the faults attributed to cotton, cotton is in reality the most permanent and easily adjusted of root fillings.

The use of cosmoline in canals is not original with me but was first suggested by Dr. St. George Elliot, of London.

Having now given you my defense of cotton dressings, let us, with your permission, proceed to consider how and when they should be inserted.

Any practical method of cleaning and sterilizing the canals can be used, but where the pulp has putressed, I invariably employ the gradual stopping process which is so clearly explained by my beloved and respected friend, Dr. Flagg. Of course, you are all familiar with it; but to keep the links in my chain of evidence perfect, with your permission I will explain his manner of treatment, which has the advantage of cleansing with equal thoroughness the small and large canals.

The tooth must first be opened and the floor of the pulp chamber so burred that the mouths of all the canals visible or invisible shall be exposed.

Then if considered practicable they can be enlarged, extreme care being taken not to puncture the cemetum.

At the first sitting all the decomposing material that can be reached should be removed. And after the passages have been thoroughly washed by streams of warm water squirted into them from the syringe, they should be dried and protected by the napkin or dam. Finally being filled loosely with a cotton dressing thoroughly soaked in pure carbolic acid, or whatever medicament may be preferred.

This, of course, should be done early in the morning. The advantage of choosing such an hour has been known to dentists for a long time. Its scientific explanation is easy.

The velocity of the blood is greatest at nine o'clock in the morning and six at night. Its ebb tide is about one P. M. and at midnight. [See Dalton's Physiology, p. 313.] This fact effectually answers all speculation on the subject. When the rapidity of the blood is increasing, any irritation will end in inflammation much more readily than when the velocity of the vital fluid is on the ebb. Therefore, judging from this fact, were we so inclined we could treat teeth with equal advantage, at six or seven P. M. But, of course, this not being feasible, the morning hour is the hour usually chosen. On the following day the syringing and replacing of the dressing should be repeated, the dressing being packed a little tighter. This should be continued each consecutive morning until twisted cotton, soaked in carbolic acid, can be firmly packed into the canals and there remain, the tooth being absolutely free from inflammation.

Extremely sensitive teeth with open canals have yielded to this treatment again and again, becoming sound and painless in a few days. Should the tooth resist the first treatment in the morning, allow cotton to rest in the canals very loosely, and tell the patient to return at eleven, when the treatment again being performed, the pain will, in almost every instance, abate.

By following this plan, any little accumulation of gas may be removed, and yet the advantage remain of treating while the blood is still on the ebb.

By this method the hair-like canals are perfectly cleansed: for the organic matter putrifying in them is each day washed out, while each cleansing is followed by an application of carbolic acid, which if the tooth is dried, will go into places inaccessible to cotton.

With these means I think you will confess that any canal, no matter how minute, can be cleaned and sterilized. This being accomplished and the last dressing allowed to remain, the tooth should be temporarily filled with gutta-percha or cement for a length of time sufficient to test the thoroughness of the work; which having been satisfactorily demonstrated the tooth may be permanently filled. The details are as follows:

Put on the rubber dam, remove dressings and blow hot air into the tooth until it becomes painful. Then, using a hypodermic syringe filled with warm carbolized cosmoline, pump the canals full.

In dealing with the large canals this will be an easy process. In those of a small diameter the passage of the cosmoline to the apex will be aided not only by capillary attraction, but also by the contraction of the cooling air. By finally pressing a pellet of cotton soaked in cosmoline over the small orifices, and then inserting a minute shred of cotton wherever possible, it seems reasonable to suppose that the canal can be filled to the apical foramen, with an antiseptic substance sufficiently viscid to exclude moisture from without. Cotton should then be packed in the large canals to act as a support for the medicament.

The canals should be filled with cotton to the pulp chamber, and a small pellet soaked in cosmoline placed over the orifices of those which are too small to allow the entrance of a thread. The cavity should now be washed with chloroform to remove superfluous grease, and the pulp chamber filled with gutta-percha or cement. I connect the mouths of the canals with protected cotton in order to expedite venting, should it be necessary. This is merely my personal preference. It is not essential. The filling to be used in covering the contents of the pulp chamber of course must vary according to the individual peculiarities of the tooth.

How is it possible for a tooth thus treated to need venting?

Because in every case there is a strong probability that the outer portion of the apical foramen may be unprotected, and, moreover, the place where the living and the dead tissues join is always a weak spot. And after all has been said and done, and the greatest care has been used, a gouty, lymphatic or plethoric patient may most unexpectedly give us a very serious example of peridontitis.

I must add, in closing, that the dentist who can always be certain of his work, in such patients, must not only be extremely sanguine, but remarkably fortunate.

This paper has been written for the purpose of showing that cotton is a legitimate root-filling. If, in the course of my argument, the disadvantages of gold, cement and gutta-percha have been brought to light, it was purely accidental.

My intention is not to prove the advocates of recognized and well tried methods unlearned, or, what is worse, unprofessionial, but to remove the stigma from the advocates of cotton dressings. No man can justly be called unprofessional, until it is clearly shown that he advocates a method contrary to the highest teaching of the day. And, before a method can be proven illegitimate, its adversaries must first clearly demonstrate that they know a process indisputably better.

These statements being accepted, it alone remains for the advocates of gold, gutta-percha and cement to prove that these materials do not have the faults attributed to cotton, and also to prove that the obvious advantages of cotton do not more than compensate for its disadvantages, whatever they may be.

All are anxious to have this question settled. If the use of cotton-fillings is bad practice, point out concisely in what respects the use of gold, gutta-percha and cement for root-filling is superior.

Let the following questions be distinctly answered:

1. Can gold be packed in the delicate canals of molars so as to exclude serum.

2. Does not gutta-percha leak, thereby allowing free entrance of putrefactive agents into the sterilized canals ?

3. Does not cement, being porous, offer similar objections. ?

4. And cannot a perfectly water-tight, easily-removable filling, be made with cotton dipped in a thirty-five per cent. mixture of carbolized cosmoline ?

DISCUSSION ON DR. HEAD'S PAPER.

*Dr. James Truman*—I do not feel any more convinced in listening to Dr. Head's paper than I was by his remarks at the Anniversary Meeting, when I expressed my opposition to cotton filling. I have some forty years behind me in the treatment of root canals, and over thirty years in which I have practiced all the various modes of filling except that with cotton. When I hear it stated that gold fillings in root canals are failures, my mind goes back to the operations of our older practitioners, Maynard, Townsend, Westcott, Dwinnelle, and others, and wonder if all their operations were failures. I used tin and gold exclusively for this purpose for many years prior to the introduction of oxy-chloride of zinc, and the cases were few where I failed to accomplish good results. We are unfortunately only too well aware that it is impossible to fill all roots perfectly, notably the buccal roots of the superior, and the anterior roots of the inferior molars; but can Dr. Head, and those who think with him, have better success with cotton? It seems to me impossible to force this into tortuous canals any better than the denser materials.

The experiment with gutta-percha in glass tubes might be conclusive if glass could be depended on. It is not a reliable material to fill against, being subject to expansion and contraction, and must eventually permit the analine to pass in at the edges. We do know clinically that gutta-percha will prevent leakage for years and years, and I think it will be difficult to show teeth better preserved than with this material. I cannot discuss the filling of canals with cotton and cosmoline. There is a possibility that cosmoline may have an advantage over carbolic acid alone, for that agent has its limitations as a germicide.

We do not want to remove a filling after it is once inserted. To fill with this idea is, to my mind, a fallacy. If we have treated the canal properly in the first instance, we want a filling placed where it will stay. I pack the gold in the canals with the mallet, and know that it will not leak there any more than it will externally.

I took the position—and the paper is an answer to one I read at the Anniversary Meeting—that before filling a canal it must be properly treated, and I further contended that no tooth was properly prepared where the canal was not free from decomposed tissue, and I stated, further, that as the dentine was made up of innumerable tubes, and that these tubuli contained organic matter, and if this material, undergoing decomposition, is not included in the treatment it becomes a constant source of danger to the tooth. I enforced the opinion that it was necessary to reduce this to an insoluble compound, and that this was best done by keeping the canal under the action of oxy-chloride of zinc. This, in my judgment, is at present the best known filling material for canals. I am aware that cotton is a good filter for micro-organism, and as long as it is not disintegrated in the canal it may be an effective agent; but when this does occur the results were exceedingly disastrous.

*Dr. J. D. Patterson, Kansas City*—Twenty years ago I had the misfortune to use cotton fillings, following the precedent of my preceptor. We did not use carbolized cosmoline as a dressing, but what was common at that time, creosote and carbolic acid. The frequent failures resulting from that practice, together with the advice and experience of others, caused me to soon discard it, and for a great many years I have employed another method. I do not wish to criticize. If it were not for the variety of opinions it would be useless for dentists to come together for discussion. I have great respect for those who take a bold stand and back their assertions with logic.

With all deference to Dr. Head, I have no confidence in the method he represents. It is no doubt better than the old method of carbolic acid alone, or better than the old English method of using cotton with sandarach. I think other methods are better in point of efficacy. There is an objection in mind to gutta-percha, so universally used throughout the West, and that is its tendency to leakage, I have found the fault present many times.

With regard to Dr. Head's experiment with the glass tube, if he will conduct one more he will have a different result. Take one of those tubes and by melting draw it into a fine thread, afterwards grinding it off. Then pack into it a gutta-percha filling up against this small opening, sealing the rest of the tube carefully; put the tube in a vessel of analine, or any other fluid, and you will have no leakage. I prefer to fill the apical portion of the canal to a small distance with gutta-percha; then the balance of the canal with oxy-chloride of zinc. In young teeth it can be forced perhaps into the dental tubuli. It is an antiseptic, and when hard renders the organic matter a fixed material. I object to filling the root entirely with oxy-chloride on account of forcing through the foramen. A great many use a solution of base plate gutta-percha. I recommend a solution of the white gutta-percha, for by experimentation I have found tbat there is not so much leakage.

*Dr. L. A. Faught*—For many years I have filled roots of teeth with cotton, and one reason for my so doing is that I have learned the value of such proceeding from both ends of the instrument. I have been a patient as well as an operator. That such fillings are as satisfactory as any others is beyond question. I have in my mouth a lower molar which has been root-filled with cotton for seventeen years, and I have yet to have the least trouble with it. If all the roots I treat give no trouble in half that time, I shall be satisfied with my results as an operator. In answer to the question:

"Why do we want to remove the filling?" I would say that if when trouble arises the patient returns to us, we can, by reference to our records, recall what has been done, and if such reference shows that what was previously accomplished was as thoroughly done as possible, then we do not want to take the filling out. Many patients, however, do not return to us, but they drift to others, or possibly when they seek us we are absent from the office, and relief is sought elsewhere. It is in such cases that—not knowing how thoroughly the treatment was originally—the operator removes the root filling so that he may be sure to place the tooth in good condition for the future. If the canal has been filled with cotton he can do the work with ease and give comfort to the suffering patient.

*Dr. Alonzo C. Boice*—Is not the whole object to prevent any leakage through the apex? The question then is what is the best method? I cannot succeed with gold, gutta-percha, nor sulphate of zinc. I find it impossible to pack them in the canals. If you take a little cotton and pass it to the end of the root it stays there, and if the canal is dry it is all right. I lately had the privilege of taking a cotton filling out of an old root of a first bi-cuspid tooth. It was sealed in with gutta-percha, the cotton being packed the length of an inch. It was in twenty-five years, and was as sweet as you could wish. It was first filled with gold, then amalgam, and finally patched up and a crown put on. In the discussion we all acknowledge that the canal must be thoroughly cleansed. If it is thoroughly prepared and dried, and you put a filling in the end of that root, will there be any trouble? I have had more success with cotton than with any other material, although I do not always have success with it. I have taken out fillings of gold that were not very pleasant.

*Dr. Bonwill*—During eight years of my earlier practice, I filled with nothing but gold and tinfoil. Sometimes I had an abscess, and I know the reason, the nerve was not thoroughly extirpated. In some small cavities it is impossible to introduce a broach, and at that time it was difficult to get a good one, as little was known about them. My knowledge of inflammation led me to experiment—to thoroughly extirpate the pulp and leave the cavity open. Many roots which have not been sterilized have given no trouble. I found that whether the pulp was treated with arsenic or not, it made but little difference, the secret of success being in the extirpation of the pulp, and when this was done it did not matter whether you filled the pulp chamber and canals or not.

After a little judgment, and some practice and experience, I commenced to experiment with cotton. The early experiments of Pasteur sufficiently justified anyone in such treatment. He took two pans of milk—one with cotton over it and one without. That which had the cotton over it would stand for days and weeks without any change. That which had nothing over it, and came in direct contact with air was soon changed. Another thing I know, cotton can be worked as thoroughly—can be packed so solidly that you cannot get moisture into it. Knowing the difficulty I had in a great many small cavities of getting gold and tin to the apical portion of the canal, and the occasional trouble I have had with them afterwards, it led me to go into the treatment with cotton, there being no trouble in packing a fibre of cotton. With it I have saved without abscess ninety-five per cent. of the cases that have come to me. It has done for me what I am perfectly satisfied I could not have done with any other material. You cannot pack gutta-percha nor get the chloroform mixture there except in a large canal. When it becomes necessary to open into any canal filled with cotton you will find it an easy operation. If filled with gold, as soon as you put the dental-engine upon it and heat it up, it will throw out gas, showing that the tooth substance has absorbed gases. Cotton cannot do any more. I can recall many instances where I put in cotton many years ago, and it has never given any trouble.

*Dr. Bennett*—Dr. Flagg, speaking of pain about devitalized teeth, calls the peridental membrane the "seat of war," and I think the same term may be not inaptly applied to root canals, as a theme for discussion. And the war still goes on apparently as far from the end as ever. But we have made progress recently as to *cleansing* and *treatment* of canals, which are after all the essentials of success. Little has been said about the sources of our knowledge, experimentation, and experience. There is always a difference in tests made in the tooth and in substances of a different nature. I have tested in glass tubes, and I cannot say I would depend as much on such tests as on those made in the teeth. The experiment shown by the essayist does not resemble a filling in a canal, but the method suggested by Prof. Patterson is something to the purpose. You can depend on a test like that every time.

If a man is successful with any method he is entitled to be heard. But then what is success? It certainly has degrees, while failure is something more definite. As to root filling, I do not think that cotton is bad under all circumstances. I would not, however, call it filling, but *treatment continued*, for do not its advocates depend on it as a "line of retreat?" And have not these same men the most to say about "retreating?" Some generals as well as operators lay more stress upon such a "line," and seem to need it more than others. The operator who fills the canals with copper amalgam must feel sure of his ground, for this, above all things, is not a "serial" filling.

I have had cases of very bad teeth that I did not venture to fill for a long time, but kept the canals full of cotton saturated with bi-chloride of mercury and iodoform. In such cases, however, it is the *medicine* and not the *cotton* that is depended on. Iodoform is the most persistent and permanent of medicines, and is therefore the best to counteract a persistent septic condition. Nearly all other medicines disappear in such cases as I referred to, odor and all, and that, too, no matter how perfectly the canal was cleansed or how well it was corked at both ends.

But filling is only one thing, and not the most difficult or important. Cleansing the canal and disinfecting or desiccating the tubuli and closing the foramen are the first three essential steps to successful treatment. If a canal must be enlarged, and I for one often find this necessary, first dry it thoroughly with hot air, so that no septic fluid be forced through. Begin with the larger drills, Morey or Gates-Glidden, and proceed gradually to the small ones. For the rest, poison and burn out the germs and gases. These, not the *filling*, are the points that count.

As to filling, I regard cotton at one extreme and gold at the other. Both are primitive materials. Cotton is easily applied and not conducive to cleanly and careful work; gold is simply a stopping, and requires skill for its insertion. Of the other two materials, gutta-percha and oxy-chloride of zinc, they are the best adapted to the greatest number of cases and operators. Gutta-percha, like gold, is merely a stopping; but when it is forced through, it is, unlike gold, non-irritating. It has been said that it is permeable by gases but what material is not? Oxy-chloride of zinc is the only material that reacts on the gases, or that has any effect on the organic portion of the dentine.

*Dr. H. C. Register*—If I understand the purport of the paper it embraces the filling of pulpless teeth under all conditions. The discussion, to my mind, refers only to the immediate filling of canals when the pulp is destroyed and removed by intent. Under such conditions I can understand how cotton can be judiciously and successfully used. If, however, the subject embraces all classes of pulpless teeth, then I think Dr. Truman takes decidedly the proper stand. To make a sweeping assertion that the cotton treatment covers these numerous conditions of pulpless teeth is to admit either ignorance or indifference on the part of the operator. If the pulp is taken away what is the object of filling the chamber? There must be some reason for it, and now let us see if we can, in our imperfect way, throw some light upon the subject.

A tooth that has its pulp destroyed by intent, retains its dentine basic substance etc., all in normal condition, less the fibrillæ, which had their origin in the pulp, and die with the source from which they received their nourishment. No degenerate action further than devitalization having taking place, it is but necessary to remove the pulp, and render the adjacent parts in condition beyond contagion of a ferment, or the sequence of bacterial action; and to this end I can understand how cotton judiciously impacted can remain pure and protect the parts for an indefinite number of years.

If, however, we leave this class of devitalized teeth for those that have passed into a putrescent state, of greater or less duration of time, we have a very different condition upon which to operate. The dentine we find is composed of about twenty-four (24) parts of organic matter, the pulp a highly organized body of organic matter only; and the death of these tissues of long standing may be divided into two classes. The one opened from the onset to the atmospheric influences. the other hermetically sealed within the long chamber. In the latter case we have degenerate matter only —broken down tissue; while in the former we have in addition to that condition of things, all the complications of micro-organisms added to it. It is these two classes of conditions that give the greatest trouble in sealing up the canals. The dentine in either case has passed from a normal condition. In the case of the open cavity, we have the basis substance, in many cases, permeated by the conditions found in ordinary caries; the tubulated structure encroached upon, oft-times the whole length of the tubuli being filled with fungus bacterial growth, in addition to inroads made into the basis substance itself; and the whole mass more or less a tooth garden of new life flourishing upon the inanimate tissue that is passing into waste. In the case where the tooth is sealed from external action, and the pulp devitalized, we have degenerate tissue only. But open these chambers to atmospheric and salivary action, and it will require but a few hours to place them in the same condition in which we find the cases just quoted. With these conditions it becomes necessary in the one case to remove the maximum amount of degenerate matter, and insure its inertness, and destroy all morbid growths found within the organ. before the canal can be filled with safety. In the latter case it is necessary only to render the degenerate tissue inert, and prevent micro-organisms from gaining a lodgement therein. Now when we focalize the object in view in the treatment of these cases, it confines itself to placing the dentine in a condition of non-activity, or otherwise suspending the action of any influence that can become an irritant. To this end do we resort to antiseptics and germicides.

The greater or less length of time, in my judgement, has little to do with its success. The agent that will suspend or control all degenerate action has everything to do with the success, whether it requires minutes, hours or days. From clinical observance, general practice and theoretical investigation, this course of treatment holds good. When the pulp is destroyed by intent and carefully removed, the dentine thoroughly desiccated, and the chamber walls coated with some germicidal agent, the tooth may be successfully filled and the pulp chamber left vacant; or, the chamber may be filled with any material that will hermetically seal it. The two latter conditions, which are typically alike, require the dentinal territory to be thoroughly robbed of its resident moisture, rendering it sponge-like, which can be done by forcing into it through a system of compressed air, heated as hot as the patient can bear it, and carried to the most distant parts of the canals by platinum hair tubes, and followed, while in a condition of thorough dryness, with a germicidal agent that will permeate it to its peripheral surface, rendering the whole territory inert. The cavity may be filled with gold saturated with oxy-chloride of zinc, or any material which will hermetically seal it. Copper amalgam, into which is rubbed a small quantity of the following dressing, has, in my hands, given me the most satisfaction.

℞. Oil Peppermint.......................................ʒj

Iodoform ....................................} āā ℥ss

Glycerine Starch..............................}

The dressing is easily carried upon a broach. The vegetable starchy matter, being in such small quantity, does not decompose, and makes it of a pleasant consistency for use. The oil of peppermint in combination with the iodoform is far-reaching and lasting.

*Dr. Tees*—For many years it has been my practice in filling single-rooted teeth easy of access, to pack a pledget of cotton saturated with carbolic acid in the apical portion of the canal and fill the remainder of the canal and pulp cavity with gutta-percha. In teeth of more than one root, I have been content to fill the opening of each canal with a pledget of cotton saturated with carbolic acid and filling the pulp cavity with gutta-percha. When this has been carefully done, and has been expedient in after years to remove it, the cotton has not been found offensive to the smell. I think it is well that the leading men of the profession are giving up gold as a canal filling. I regard oxy-chloride of zinc as an excellent filling for that purpose, and I endorse Dr. Truman's treatment so far, preferring, however, the placing of the cotton and carbolic acid in the apical portion of the canal.

*Dr. Dean*—I would like to ask Dr. Register if he cleans out the root of all its dead material?

*Dr. Register*—I strongly advocate cleansing out all the tissue from the root canal. At the same time I think the success is largely due to cleansing the mouths of the tubuli, and at the same time preventing any entrance of micro-germs. I think the success is in cleansing out the canal and preventing any degenerate condition.

*Dr. Head*—I would like to reply to Dr. Truman in relation to gutta-percha. He said that gutta-percha has a good record for preserving tooth structure. This I do not deny, but still affirm that it absorbs fluids and that, therefore, is an imperfect canal filling. Dr. Patterson's objection to my test in the glass tube is, of course, perfectly legitimate; but I think this test has been carried out more accurately than he supposes. Some years ago I came upon a tooth that had been thoroughly treated and thoroughly filled with gutta-percha to the end of the canal. There was an alveolar abscess, but the gutta-percha was permeated from apex to pulp cavity with gas. But in all this discussion no one has answered any of my questions. I will ask Dr. Truman, does not gutta-percha leak?

*Dr. Truman*—I do not think so.

*Dr. Head*—I leave his answer with you. Dr. Truman, does not oxy-chloride of zinc absorb fluid?

*Dr. Truman*—It does.

*Dr. Head*—Then, if oxy-chloride of zinc absorbs fluids, how can this material protect a sterilized canal from putrefactive germs? How can it possibly be called a perfect filling?

*Dr. Kirk*—The method described by Dr. Head of filling root canals with cotton, armed with carbolized cosmoline, is, in my judgment, not in any sense an argument in favor of cotton as a root filling, but one in favor of *cosmoline*. The cotton is merely incidental, in the same way that it is often used in connection with oxy-chloride of zinc, viz.: as a vehicle for carrying to place the real filling material, which is the cement. Cosmoline is a heavy hydro-carbon oil, totally unalterable in air or moisture by virtue of its non-affinity for oxygen. It is sufficiently viscid to remain in the canal almost without the help of the cotton, which, in its relation to the cosmoline, fulfills the same function as the old root filling of gold saturated with gutta-percha solution.

In its general characteristics, a root filling of cosmoline and cotton would be very similar to that of paraffine, which is exceedingly valuable, and can be pumped in a melted state into the finest canal—with the advantage that when it is chilled and solidified it is denser. Subject passed.

